# Development and implementation of a student tumor board as a teaching format for medical students

**DOI:** 10.1007/s00432-023-05336-3

**Published:** 2023-09-12

**Authors:** Irina Mäurer, Robert Drescher, Jakob Hammersen, Nora Dieckmann, Yvonne Gremme, Max-Johann Sturm, Aaron Lawson McLean, Anna C. Lawson McLean, Christian Senft, Andrea Wittig, Caroline Klingner, Christiane von Sass, Matthias Mäurer, Marcel A. Kamp

**Affiliations:** 1 Department of Neurology, Jena University Hospital, Friedrich-Schiller-University Jena, Am Klinikum 1, 07747 Jena, Germany; 2 Neuro-Oncological Center, Jena University Hospital, Friedrich-Schiller-University Jena, Jena, Germany; 3 Advanced Clinician Scientist Program “AntiAge”, Jena University Hospital, 07747 Jena, Germany; 4 Department of Nuclear Medicine, Jena University Hospital, Friedrich-Schiller-University Jena, Jena, Germany; 5 Department for Haematology and Medical Oncology, Jena University Hospital, Friedrich-Schiller-University Jena, Jena, Germany; 6 Department of Radiation Oncology, University Medical Center Jena, Hospital, Friedrich-Schiller-University Jena, Am Klinikum 1, 07747 Jena, Germany; 7 Department for Neurosurgery, Jena University Hospital, Friedrich-Schiller-University Jena, Jena, Germany; 8 Centre for Palliative Care and Neuropalliative Care, Brandenburg Medical School Theodor Fontane, Campus Rüdersdorf, Seebad 82/83, 15562 Rüdersdorf, Germany; 9 Faculty of Health Sciences Brandenburg, Brandenburg Medical School Theodor Fontane, Institute for Health Services and Health System Research, Campus Rüdersdorf, Seebad 82/83, 15562 Rüdersdorf, Germany; 10 Clinician Scientist Program “OrganAge”, Jena University Hospital, 07747 Jena, Germany

**Keywords:** Neuro-oncology, Tumor board, Flipped classroom, Medical education, Teaching format

## Abstract

**Purpose:**

Tumor boards serve as established platforms for interdisciplinary expert discussions and therapeutic recommendations tailored to individual patient characteristics. Despite their significance, medical students often lack exposure to such interdisciplinary discussions as tumor boards are currently not integrated into medical curricula. To address this, we aimed to enhance future physicians' interdisciplinary communication skills and subject-specific knowledge by introducing an interactive series of five linked tumor board seminars within the domain of neuro-oncology.

**Methods:**

We developed a neuro-oncological student tumor board using a flipped-classroom format. The primary objectives of this case-centered approach included fostering an understanding of the tumor board process, active participation in multidisciplinary case discussions, honing appropriate communication strategies, and creating personalized therapy plans that consider inputs from all relevant disciplines, individual patient factors, and ethical considerations. To gauge the effectiveness of the seminar series, we administered structured pre- and post-course questionnaires.

**Results:**

Fourteen medical students in third to fifth year participated in the pilot series. Despite its organizational complexity, the interdisciplinary seminars were feasible. Students demonstrated significant growth in competence, aligned with predefined learning objectives. Notably, they appreciated the supportive learning environment and interactive teaching format, which kindled their interest in interdisciplinary oncology.

**Conclusion:**

Active participation in a student tumor board can empower students to tackle the diverse challenges of caring for cancer patients within an interdisciplinary team during the early stages of their careers. The student tumor board represents an innovative, learner-centered approach to teach interdisciplinary cancer treatment, communication strategies, and ethical aspects of medical practice.

**Supplementary Information:**

The online version contains supplementary material available at 10.1007/s00432-023-05336-3.

## Introduction

Modern personalized oncological treatment concepts require interdisciplinary and multi-professional collaboration (Soukup et al. 2018; Selby et al. 2019). The development of individual therapy plans for cancer patients must be based on scientifically evaluated and evidence-based sources (Schirrmacher et al. 2023). In the absence of such sources, therapy recommendations should be based on best medical practice. Currently, complex therapy concepts for cancer patients are not formulated by individual specialists but by teams of experts from various disciplines. The performance of these well-coordinated specialist teams surpasses the individual contributions of each team member (Mäurer et al. 2022).

Interdisciplinary case reviews, also known as *tumor boards*, provide the panels of for specialists from different disciplines to collectively develop treatment recommendations for individual patients. These interdisciplinary discussions with creation of individualized therapy plans are independent quality indicators and essential requirements for successful certification as oncology centers (Brucker et al. 2009; Griesshammer et al. 2023; Roessler et al. 2022). Discussions of oncological patient cases in an interdisciplinary setting encourage evidence-based treatment and often lead to a change in diagnostic procedures or therapeutic management, potentially improving patient outcomes (Munro et al. 2015; Pillay et al. 2016; Algwaiz et al. 2020). Moreover, effective case discussion requires thorough preparation and focused knowledge of the literature, as well as research by the involved physicians. Apart from evidence-based therapy plans, it is equally important to consider patients' wishes, ideas, needs, and expectations. Additionally, oncological treatment raises numerous ethical issues that require consideration (Kuroki et al. 2010).

Medical schools are responsible to adequately prepare and equip students for the requirements of their future profession (Frank et al. 2015). Students rely on acquiring the necessary competencies to meet professional demands and fulfill the various roles required of physicians (McGaghie et al. 2011). These roles encompass being amongst others a medical expert, a member of an interdisciplinary and multi-professional team, a communicator, a health advisor and an advocate who encourages patients and their families to deal with life-threatening conditions (Frenk et al. 2010). Integrating the acquirement of these different competencies into medical education is crucial for future physicians to empower them to deliver excellent patient-centered care that accounts for unique needs of each individual.

To date, mandatory participation in interdisciplinary tumor boards is not foreseen in the curricula of many medical faculties. There is a lack of conceptual foundations to teach medical students the key skills necessary for successful participation in interdisciplinary case discussions. These specifically include interdisciplinary communication, presentation skills, and understanding of joint benefit-risk assessments. As a result of this weakness in the training of medical students, young physicians may struggle with the mechanisms and procedures of an interdisciplinary tumor board in their professional routine.

From our perspective, the sub-specialty of neuro-oncology is particularly suitable for teaching medical students the above-mentioned key skills (Mäurer et al. 2022). Neuro-oncology encompasses several disciplines including hemato-oncology, neurosurgery, neurology, and radiation oncology together with a high involvement of further organ-specific specialties, such as dermatology, gynecology and urology. Intensive interaction with palliative care medicine, psycho-oncological support and trained nursing staff makes neuro-oncology a highly multifaceted and multi-professional medical field. Ethical aspects of oncological practice are particularly relevant in this field, given the tension between prolonging life and maintaining quality of life, and considering the presumed will of patients who are unable to consent (Doukas et al. 2015).

Therefore, we have established a neuro-oncological student tumor board to teach medical students how a tumor board functions including interdisciplinary communication mechanisms, the weighting of pros and cons, evidence-based therapy plan creation, discussion of ethical issues and consideration of individual patients' wishes and needs.

## Material and methods

### Ethics approval

The investigation adhered to the ethical principles outlined in the 1964 Helsinki Declaration and its later amendments. All procedures involving human participants in this study were approved by the institutional and local ethics committee (study ID: 2023–2912-Bef, ethics committee of the Jena University Hospital, Germany).

#### Learning objectives

We defined the following major learning objectives:

1. Active participation in interdisciplinary case discussions using effective communication strategies
2. Development of multimodal therapy plans in an interdisciplinary team under specialist supervision and guidance
3. Consideration of the individual risk factors, pre-existing conditions and their patients’ perspective and their social situation
4. Efficient use of scientific and other evidence-based sources to create individualized contemporary treatment plans
5. Consideration of medical interventions and oncological treatments in combination with ethical aspects

#### Course format and structure

In accordance with the constructive alignment method developed by John Biggs, we aligned both the teaching and examination settings with the learning objectives (Biggs 1996). An important learning goal is to empower and enable students to independently create interdisciplinary therapy plans in a student tumor board. At this early stage of the medical training, students usually do not have the required specialist knowledge. In order to promote active learning of related factual knowledge, we integrated the student tumor board into a flipped classroom format (O'Flaherty and Phillips 2015). We provided essential information on evidence-based knowledge acquisition, communication strategies, epidemiology, pathology, diagnostics, therapy and prognosis of neuro-oncological conditions via Moodle (version 3.11; https://moodle.org, Moodle Pty Ltd., Australia). During in-person sessions, the students were introduced to the consultant supervisors of the involved disciplines, the student tutors, and the context of the seminar. Students were assigned specific clinical roles, such as medical oncologist, neurologist, neurosurgeon, radiation oncologist, and neuro-radiologist. Additionally, we provided seven representative case vignettes for interdisciplinary discussions. Each case vignette contained one pre-tumor board and one post-tumor board section, enabling students to compare their final treatment decision with a similar real-world case scenario during the fifth session. Following a four-week self-study phase with the online learning materials, participants simulated an in-person student tumor board. Based on the provided case vignettes, participating students created therapy plans in the tumor board simulation setting without active intervention by the observing consultant supervisors.

#### Target group and curriculum implementation

**Fig. 1:**
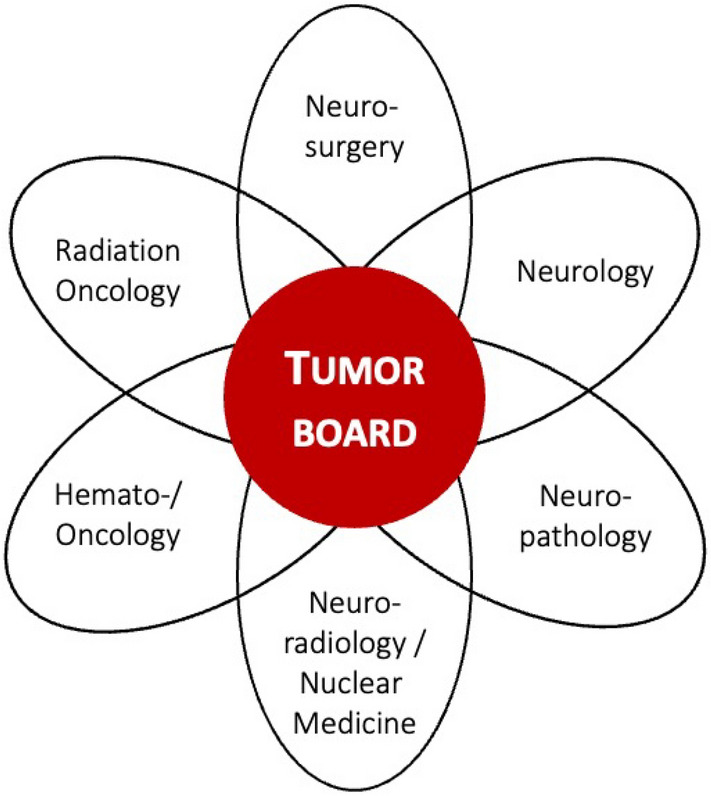
Mandatory participants in neuro-oncology tumor boards according to the accreditation guidelines of the German Cancer Society for neuro-oncology centers. Neuropathology was not included in our tumor board seminar in the pilot phase. Associated disciplines such as palliative medicine, gynecology, dermatology, etc. can be invited as needed

This course targeted medical students in their clinical training (3th to 5th medical year), with a planned group size of 5–12 students per course. In the pilot phase, we offered the event twice, with a total of 14 students from the 2nd to 4th clinical semester participating in the seminar. The mean age was 23 ± 0.9 years, and eight students were female. The students were enrolled in the student neuro-oncological tumor board to represent the disciplines required for a representative simulation. A predetermined interdisciplinary clinicians group comprising a neurologist, neurosurgeon, radiation oncologist, (hemato-) oncologist, and neuroradiologist continuously taught and supervised the students in all course-related aspects (Fig. 1).

Initially designed as a hybrid format with only two in-person sessions, the seminar structure was adapted to five on-site sessions based on students' agreement when COVID regulations were relaxed at the start of the seminar series. The seminar format is also suitable for a virtual tumor board version where necessary. The course was initially offered as an elective seminar series within our institution's curriculum, with the possibility of becoming an integral part of medical training for all students if positively evaluated. Moreover, we aim to adapt the teaching format to other oncological sub-disciplines.

#### Evaluation and assessment of success

To evaluate the impact and success of our project, we conducted a scientific validation by means of a modular, standardized questionnaire. The questionnaire was based on the guidelines for evaluating student courses developed by the Humboldt University Berlin (Braun et al. 2008; Qualitätsmanagement 2019). The questionnaire was pseudonymized to enable collection of socio-demographic information from the students, assessment of teaching and learning success concerning the predefined teaching goals, communication and interaction in the course, as well as the assessment of the tutors (for the complete questionnaire, see Supplemental File I. An English translation can be found in Supplemental File II).

At the beginning of the course, we collected basic data and assessed students' familiarity with the discipline of neuro-oncology and relevant interdisciplinary soft-skills. At the end of the seminar, we performed a paper-based comparative assessment of teaching and learning success. Additionally, after each teaching session, we obtained brief feedback from the students using various digital and in-presence methods, such as one-minute papers and flashlights.

#### Statistical analysis

For the statistical analyses and graphs, we used Graph Pad Prism 9 for macOS (Version 9.5.0, GraphPad Software, Inc., La Jolla, USA). We only considered fully completed questionnaires for further analysis, and present continuous data as means and standard error of mean (means ± SEM). We show categorical data as frequencies and percentages. As the number of participants during the pilot phase was small, we did not determine any correlations.

## Results

### Implementation and process of the student tumor board

**Fig. 2:**
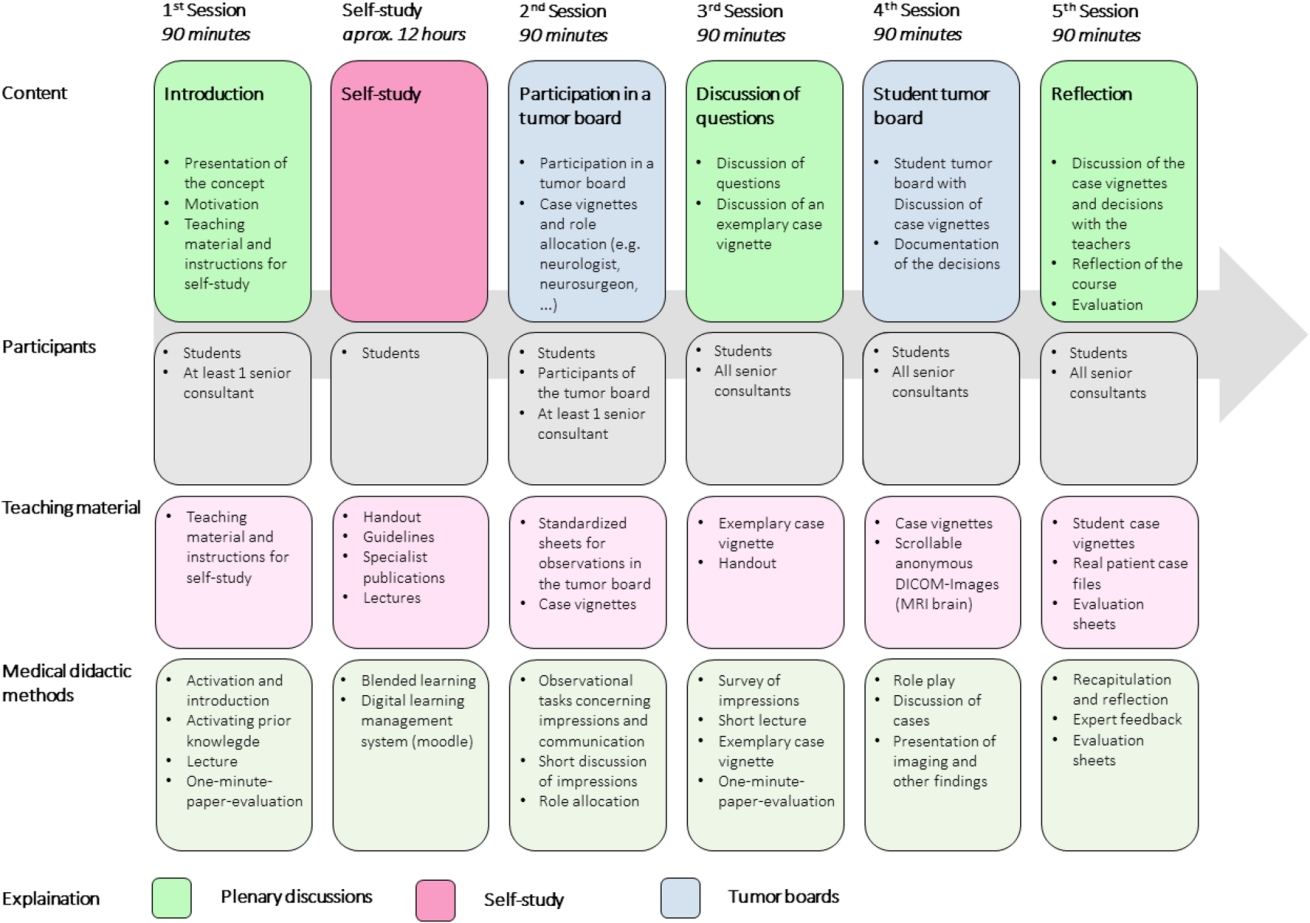
Structure of the seminar. The figure shows the structure of the seminar series comprising an introduction, self-learning phase, participation in a real tumor board, clarification of open questions, student tumor board with case discussions by the students and a final evaluation

To foster the ability to create oncological therapy concepts within an interdisciplinary team, we opted for a student tumor board as the setting for our teaching via the flipped classroom format to facilitate the required subject-related knowledge (Fig. 2).

During the first plenary seminar out of the five face-to-face teaching sessions, we encouraged students to participate actively in the course. Additionally, we explained the background and motivation of the interdisciplinary teaching project together with the organizational framework and further highlighted legal aspects relevant to oncological center certification. We also discussed the focal points of the 4-week self-study period with the participants. Here, we referred to the online teaching materials provided, including handouts on topics such as WHO classification of brain tumors, gliomas, meningiomas, brain metastases, neurinomas; current guidelines; selected publications and lectures on neurology, imaging, radiation oncology and neurosurgery. The consultant supervisors explained issues such as access to the Moodle learning platform and outlined the application approaches with regard of different learning material types.

In the second seminar session, the students participated in a real in-house neuro-oncological tumor board, where they were assigned specific specialist roles and asked to evaluate the process and communication between the specialists using a standardized evaluation sheet (Lumenta et al. 2019). The students received seven case vignettes to be discussed during the student tumor board session and specific specialist roles for the student tumor board were assigned to each student.

The third session provided an opportunity for students to clarify any open issues and engage in a practical discussion about a representative cancer patient case with fellow students and supervisors. The consultant supervisors of the involved disciplines presented their individual preparation strategies regarding discipline-specific perspectives and essential aspects of the organizational tumor board structure.

**Fig. 3:**
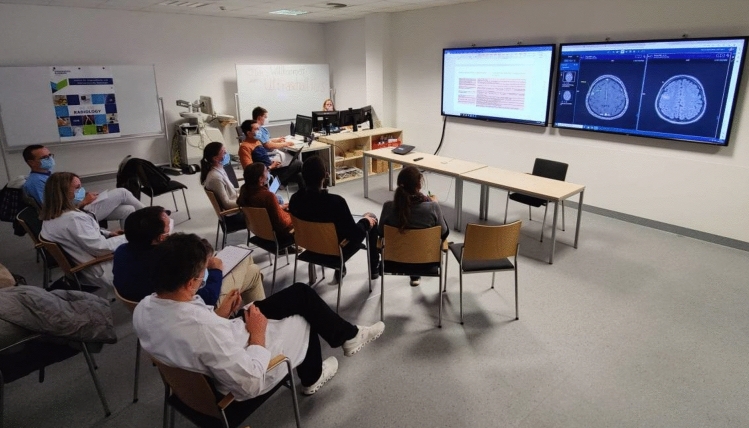
Student tumor board scenario. Practical setting of the student tumor board session (4th session). Students (first row) are engaged in interdisciplinary discussion of cancer patient cases, whose images are presented by the student “radiologist”. The consultant supervisors (second row) observe and tale notes but are not actively involved. Seminar participants engaged in interdisciplinary discussion of cancer patient cases. Student tutors and advisory supervisors accompanied and observed the discussions without unsolicited interaction

During the student tumor board session (fourth session), students discussed the provided case vignettes from the perspective of their assigned role with the assistance of the student tutors (Fig. 3). The students jointly answered questions about the further diagnostic procedures and therapy recommendations, with the consultant supervisors available for questions but not actively intervening in the communication process. A student tutor documented the students' treatment recommendations in a standardized matrix, which is the same used in the regular tumor boards of the hospital.

In the fifth session, students reported their experiences during the student tumor board session and engaged in self-assessment. The group conducted an in-depth discussion regarding the treatment decisions made during the student tumor board session, with input from supervisors and professional feedback. They discussed post-tumor board sections of the case vignettes, exploring alternative treatment scenarios. The seminar evaluation and feedback round using the flashlight format concluded the session.

#### Student population and evaluation

**Fig. 4:**
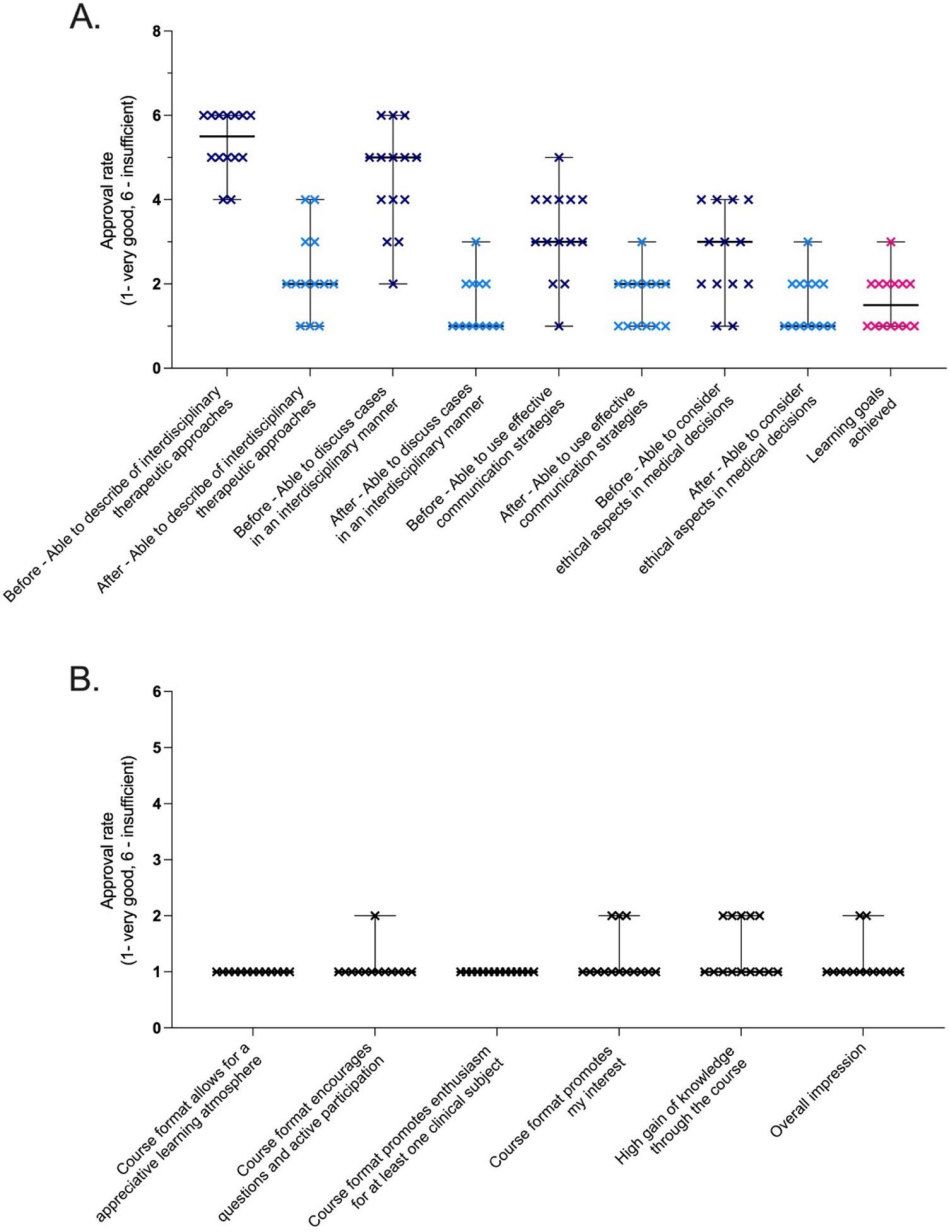
Seminar evaluation and self-assessment based on the 6-point-Likert-Scale. Students assessed their own competencies with regard to the learning objectives before and after the course (**A**). They considered the teaching format to be well suited to create an appreciative learning atmosphere, to interact, to spark interest and to convey the learning goals (**B**)

The 14 participating students devoted an average of 4 ± 3 h per week for self-study and seminar preparation. During the student tumor board, students discussed seven case vignettes in 90 min and proposed interdisciplinary therapy recommendations, aligning with those of the consultants for all 7 provided cases. At the end of the seminar, students reported a noticeable gain of competency in all predefined learning objectives (Fig. 4A). Additionally, they praised the teaching format for creating an appreciative learning atmosphere, facilitating interaction, sparking interest, and effectively conveying the learning goals (Fig. 4B).

## Discussion

The pilot phase of the described teaching format lead to the following main conclusions: (1) A neuro-oncological student tumor board is a suitable option to introduce neuro-oncology and associated disciplines in an constructive manner at an early stage of medical training. (2) The course imparts an interdisciplinary mindset, effective communication strategies, oncological decision making in an interdisciplinary team and teaches the importance of considering ethical and social issues in treatment decisions. (3) The problem-oriented learning format is feasible, can be converted to a completely virtual format and adapted to other educational fields. (4) The interactive, case-centered format fosters an appreciative learning atmosphere, generates interest in oncological disciplines, and provides students with a forum to practice interdisciplinary communication skills in a supportive environment.

Modern adult educational concepts aim at empowering individuals and imparting competencies rather than just transferring knowledge (Epstein and Hundert 2002). In this context, the teaching of competencies comprises the integration of knowledge, skills and attitudes required for successful and responsible problem-solving in different situations and focuses on individuals with specific knowledge levels, interests and capabilities. (Epstein and Hundert 2002).

In the current reform efforts of medical teaching in Germany, which are reflected in the "Masterplan Medizinstudium 2020" and in the revision of the National, there is a concrete demand for a consistent practice and competence orientation of teaching with a special focus on the understanding of an interdisciplinary effective cooperation of the different professions in the health care system(MFT Medizinischer Fakultätentag 2021; Bundesministerium für Forschung und Bildung 2020). In the model curriculum proposed by Dapper et al. visits to "real" tumor boards are suggested as elective teaching formats. Since the series event we have developed includes participation in a real tumor board, this directly addresses the aspect of practical learning experience. Whether as an elective course or as a mandatory curricular teaching format, participation in a real tumor board should be part of the basic educational training for all medical students.

In the context of the increasing complexity across all medical sub-disciplines, and oncology in particular, interdisciplinary cooperation and the development of nuanced treatment concepts represent crucial skills for future physicians (Lamb et al. 2011). Interdisciplinarity is now considered an essential necessity in medical care and a quality criterion, not limited to tertiary care centers (Brannstrom et al. 2015; Winters et al. 2021). The demand for interdisciplinary cooperation is not a new aspect (Hall and Weaver 2001; Singleton and Green-Hernandez 2023), however, the corresponding competencies have not yet been anchored in the curricula of many medical faculties. Previously published literature has repeatedly emphasized the need for the transfer of competencies for interdisciplinary cooperation (Mäurer et al. 2022; Ha and Parakh 2018; Kamp et al. 2021; Mann et al. 1996; Williams et al. 2002; Gerlach et al. 2023; Karsai et al. 2011). Nevertheless, there are almost no concepts described that convey interdisciplinary cooperation, particularly in student tumor board simulations. The assignment of specific clinical roles and case discussions of breast cancer patients were already implemented in the 1990s to show psychosocial aspects of oncological diseases. In addition, role-playing is occasionally used in other areas of medicine, such as palliative medicine or geriatrics, to practice interdisciplinary and multi-professional interaction (Williams et al. 2002). Role-playing games are used more frequently to mediate doctor-patient communication, especially to simulate the breaking of bad news (Colletti et al. 2001; Cushing and Jones 1995; Rosenbaum and Kreiter 2002). Other interdisciplinary teaching programs for students convey communication competencies, including concepts such as shared decision making, de-escalation or breaking bad news in an interdisciplinary team or multidimensional treatments (e.g., in palliative care) (Gerlach et al. 2023; Bachmann et al. 2013, 2017). However, they do not address mechanisms of interdisciplinary communication, or the creation of a patient-centered therapy plan.

Our teaching format is an approach that could rouse the interest in multidisciplinary fields, such as neuro-oncology, with reference to imparting and practicing the skills of interdisciplinary cooperation and therapy plan creation, as well as for discussing relevant ethical aspects.

We see numerous advantages of the format presented herein: (1) The seminar structure facilitates the teaching and practical application of all competencies defined by the learning objectives, while the teaching materials support independent problem-based learning. (2) The format is highly authentic and allows students to focus on relevant facts for individual patient cases and learn to manage their time resources wisely. (3) Practical implementation, as demonstrated in our pilot phase, is feasible. (4) The feedback from the pilot phase shows good results, especially with regard to the achievement of the learning goals, the enthusiasm for at least one specialist discipline and the appreciative learning atmosphere. However, the evaluation of our cohort is not significant due to its small number and requires validation by further participating student groups. Furthermore, a selection bias must be taken into account, which could have distorted the evaluation results, since the participating students may have been particularly interested in the topic and showed above-average motivation. (5) The format can be easily adapted as needed: The student tumor board need not be limited to the flipped-classroom format but can be used as part of a series of lectures or seminars to monitor learning success. Interdisciplinary communication strategies can also be integrated in other formats, such as seminars with practical exercises or as part of crew resource management (Mäurer and Interdisziplinäre tumorkonferenz. 2023; Maurer et al. 2023). (6) The learning format could arouse interest in oncology or even help to recruit residents for oncological disciplines. Through the identification of the participants with their own role, the interest in the respective discipline could increase. The extent to which students undertake an internship in this discipline (clinical traineeship, internship year) or even start their professional career in this field could be the purpose of future assessments. (7) The course format can be applied to other oncological or medical disciplines and adapted to other fields where multi-professional solution-oriented approaches are critical for successful teamwork.

We acknowledge the following limitations and challenges: (1) Our teaching format required significant time and resources. Several clinical specialists were involved throughout the course, along with various student tutors. This input was disproportionately large compared to the number of students who participated in the pilot phase. Additionally, new teaching materials, including presentations and videos, had to be designed and approved by all involved disciplines in preparation for the course. (2) The feasibility of the format depends, among other things, on the availability of preparatory courses in the field of neuro-oncology. An analysis of radiation oncology teaching at medical faculties in Germany showed that only 2/3 of all curricula cover this topic (Oertel et al. 2020). Although the basic principle of our teaching format is easily applicable to other oncological subfields, the heterogeneous training in the field of neuro-oncology limits the transferability to other faculties. (3) The flipped classroom format, described and introduced several years ago, is now well established (Lage et al. 2000). There is a potential risk of rejection of the flipped classroom format by individual participants. In our pilot student cohort, not a single student expressed criticism of the format, but nevertheless the lack of or inadequate preparation of the teaching materials provided in advance could impair learning success. We countered this risk by motivating the students and explaining the need for preparation before engaging in the interactive exchange with regard to joint learning success of the group. Although self-study can save on the teaching of basic knowledge, adequate motivation of individual participants becomes more important, as inadequate preparation can impair learning success. Based on our experience from the pilot phase, we recommend that students engage in discussions with their assigned consultant with regard to any arising questions related to the case vignettes. This approach can provide the students with additional confidence and ensure the success of the student tumor board. An additional collaborative self-study learning session through dyadic peer-to-peer interaction could potentially enhance the learning effects. (4) There is no clear consensus on the ideal timing of an interdisciplinary educational intervention. (5) Future research must determine whether conveying the concept of interdisciplinarity provides young medical professionals with the ability to participate more effectively in interdisciplinary treatment discussions and gain more confidence to participate in tumor board conferences.

## Conclusion

Student tumor board seminars, as presented in this study, have demonstrated feasibility and effectiveness in imparting interdisciplinary competencies. However, it is important to acknowledge that these seminars can be resource-intensive in terms of personnel and time. The concept of student tumor boards, where students assume medical professional roles, represents an innovative approach to teach interdisciplinary communication strategies, practice creating therapy plans, and engage in discussions about ethical issues in medical practice. Moreover, the interactive nature of simulating specialist roles in the seminar setting may empower future physicians to communicate more effectively with patients and gain confidence in navigating professional interdisciplinary teamwork.

### Supplementary Information

Below is the link to the electronic supplementary material.

Supplementary file1 (DOCX 33 KB)

Supplementary file2 (DOCX 32 KB)

## Data Availability

The data sets created and/or analyzed as part of the study can be obtained from the author upon reasonable request.
