# Supplementary material for: Development and implementation of a student tumor board as a teaching format for medical students
Source: J Cancer Res Clin Oncol. 2023 Sep 12;149(17):16087–96. doi: 10.1007/s00432-023-05336-3 (PMC10620267; doi:10.1007/s00432-023-05336-3)
Supplement: Supplementary file 1 — Supplementary file1 (DOCX 33 KB) [file 432_2023_5336_MOESM1_ESM.docx]

# EVALUATION: STUDENTISCHES TUMORBOARD

Vor dem Kurs

| Geschlecht | | | | | | ☐m ☐w ☐d | | | | | | |
| --- | --- | --- | --- | --- | --- | --- | --- | --- | --- | --- | --- | --- |
| Alter | | | | | | ________Jahre | | | | | | |
| Fachsemester | | | | | | ________Fachsemester | | | | | | |
| Ich habe den Kurs gebucht, weil *(Mehrfachauswahl)*:  ☐ es hier viele C-Punkte gibt  ☐ der Kurs mein Interessengebiet widerspiegelt  ☐ ich Kommunikationsstrategien erlerne  ☐ es eine Präsenz-Veranstaltung ist  ☐ diese Fähigkeiten anderweitig nicht gefördert werden  ☐ es eine interaktive Veranstaltung ist  ☐ ich die Dozent:innen kenne | | | | | | | | | | | | |
| Was erwartest du von dem Kurs?  _________________________________________________________________________  _________________________________________________________________________ | | | | | | | | | | | | |
| Was wäre ein No-Go in diesem Kurs?  _________________________________________________________________________ | | | | | | | | | | | | |
| Wie viel Zeit wirst du voraussichtlich wöchentlich investieren, um diesen Kurs vorzubereiten? *(Stunden)* | | | | | | | | | | | | |
| ☐ 0 | **☐ 1** | **☐ 2** | | **☐ 3** | | | **☐ 4** | | **☐ 5** | | **☐ 6** | |
| Ich habe schon vorher ein Tumorboard besucht | | | | | | | **☐ ja ☐ nein** | | | | | |
| Bitte schätze deine Vorkenntnisse in den Fächern ein: | | | **1 – sehr gute Kenntnisse** | | **2 – gute Kenntnisse** | | | **3 – befriedigend** | **4 – ausreichend** | **5 – mangelhaft** | | **6 - ungenügend** |
| Neurologie | | | **☐** | | **☐** | | | **☐** | **☐** | **☐** | | **☐** |
| Neurochirurgie | | | **☐** | | **☐** | | | **☐** | **☐** | **☐** | | **☐** |
| Strahlentherapie | | | **☐** | | **☐** | | | **☐** | **☐** | **☐** | | **☐** |
| Onkologie | | | **☐** | | **☐** | | | **☐** | **☐** | **☐** | | **☐** |
| Radiologie | | | **☐** | | **☐** | | | **☐** | **☐** | **☐** | | **☐** |
| Nuklearmedizin | | | **☐** | | **☐** | | | **☐** | **☐** | **☐** | | **☐** |

| Schon vor dem Kurs… | 1 – stimme ich vollkommen zu | 2 – stimme ich überwiegend zu | 3 – stimme ich  eher zu | 4 – stimme ich  eher nicht zu | 5 – stimme ich größtenteils  nicht zu | 6 – stimme ich überhaupt  nicht zu |
| --- | --- | --- | --- | --- | --- | --- |
| konnte ich den Begriff „Tumorboard“ einordnen. | **☐** | **☐** | **☐** | **☐** | **☐** | **☐** |
| konnte ich das Ziel und den Ablauf einer interdisziplinären Tumorkonferenz darlegen. | **☐** | **☐** | **☐** | **☐** | **☐** | **☐** |
| konnte ich die Vor- und Nachteile einer Tumorkonferenz werten | **☐** | **☐** | **☐** | **☐** | **☐** | **☐** |
| konnte ich Grundlagen zu neuroonkologischen Entitäten erläutern. | **☐** | **☐** | **☐** | **☐** | **☐** | **☐** |
| Konnte ich interdisziplinäre Therapieansätze von ZNS-Neoplasien beschreiben | **☐** | **☐** | **☐** | **☐** | **☐** | **☐** |
| Konnte ich Fälle interdisziplinär diskutieren | **☐** | **☐** | **☐** | **☐** | **☐** | **☐** |
| Habe ich effektiv kommuniziert | **☐** | **☐** | **☐** | **☐** | **☐** | **☐** |
| Habe ich ethische Aspekte in ärztlichen Entscheidungen berücksichtigt | **☐** | **☐** | **☐** | **☐** | **☐** | **☐** |
| konnte ich eine Konsensus-Entscheidung fach- und patientengerecht erläutern | **☐** | **☐** | **☐** | **☐** | **☐** | **☐** |
| Hatte ich Interesse an den teilnehmenden Fächern (Strahlentherapie, Neurochirurgie, Onkologie, Pathologie, Radiologie) | **☐** | **☐** | **☐** | **☐** | **☐** | **☐** |

# EVALUATION: STUDENTISCHES TUMORBOARD

Nach dem Kurs

| Geschlecht | | | | | | ☐m ☐w ☐d | | | | | | |
| --- | --- | --- | --- | --- | --- | --- | --- | --- | --- | --- | --- | --- |
| Alter | | | | | | ________Jahre | | | | | | |
| Fachsemester | | | | | | ________Fachsemester | | | | | | |
| Welche Erwartungen haben sich an den Kurs erfüllt?  _________________________________________________________________________  _________________________________________________________________________ | | | | | | | | | | | | |
| Welche Erwartungen haben sich nicht erfüllt?  _________________________________________________________________________ | | | | | | | | | | | | |
| Was war ein No-Go in diesem Kurs?  _________________________________________________________________________ | | | | | | | | | | | | |
| Wie viel Zeit hast du ungefähr wöchentlich investiert, um diesen Kurs vorzubereiten? *(Stunden)* | | | | | | | | | | | | |
| ☐ 0 | **☐ 1** | **☐ 2** | | **☐ 3** | | | **☐ 4** | | **☐ 5** | | **☐ 6** | |
| Für welches Fach hast du am meisten Zeit investiert?  ___________________________________________________________________ | | | | | | | | | | | | |
| Bitte schätze deine Kenntnisse in den Fächern ein: | | | **1 – sehr gute Kenntnisse** | | **2 – gute Kenntnisse** | | | **3 – befriedigend** | **4 – ausreichend** | **5 – mangelhaft** | | **6 - ungenügend** |
| Neurologie | | | **☐** | | **☐** | | | **☐** | **☐** | **☐** | | **☐** |
| Neurochirurgie | | | **☐** | | **☐** | | | **☐** | **☐** | **☐** | | **☐** |
| Strahlentherapie | | | **☐** | | **☐** | | | **☐** | **☐** | **☐** | | **☐** |
| Onkologie | | | **☐** | | **☐** | | | **☐** | **☐** | **☐** | | **☐** |
| Radiologie | | | **☐** | | **☐** | | | **☐** | **☐** | **☐** | | **☐** |
| Nuklearmedizin | | | **☐** | | **☐** | | | **☐** | **☐** | **☐** | | **☐** |
|  | | |  | |  | | |  |  |  | |  |

| Nach dem Kurs… | 1 – stimme ich vollkommen zu | 2 – stimme ich überwiegend zu | 3 – stimme ich  eher zu | 4 – stimme ich  eher nicht zu | 5 – stimme ich größtenteils  nicht zu | 6 – stimme ich überhaupt  nicht zu |
| --- | --- | --- | --- | --- | --- | --- |
| kann ich den Begriff „Tumorboard“ einordnen. | **☐** | **☐** | **☐** | **☐** | **☐** | **☐** |
| kann ich das Ziel und den Ablauf einer interdisziplinären Tumorkonferenz darlegen. | **☐** | **☐** | **☐** | **☐** | **☐** | **☐** |
| kann ich die Vor- und Nachteile einer Tumorkonferenz werten | **☐** | **☐** | **☐** | **☐** | **☐** | **☐** |
| kann ich Grundlagen zu neuroonkologischen Entitäten erläutern. | **☐** | **☐** | **☐** | **☐** | **☐** | **☐** |
| Kann ich interdisziplinäre Therapieansätze von ZNS-Neoplasien beschreiben | **☐** | **☐** | **☐** | **☐** | **☐** | **☐** |
| Kann ich Fälle interdisziplinär diskutieren | **☐** | **☐** | **☐** | **☐** | **☐** | **☐** |
| kann ich effektiver kommunizieren | **☐** | **☐** | **☐** | **☐** | **☐** | **☐** |
| berücksichtige ich eher ethische Aspekte in ärztlichen Entscheidungen | **☐** | **☐** | **☐** | **☐** | **☐** | **☐** |
| kann ich eine Konsensus-Entscheidung fach- und patientengerecht erläutern | **☐** | **☐** | **☐** | **☐** | **☐** | **☐** |
| Habe ich Interesse an den teilnehmenden Fächern (Strahlentherapie, Neurochirurgie, Onkologie, Pathologie, Radiologie) | **☐** | **☐** | **☐** | **☐** | **☐** | **☐** |
| Wie sehr stimmst du folgenden Aussagen zu? | **1 – stimme ich vollkommen zu** | **2 – stimme ich überwiegend zu** | **3 – stimme ich**  **eher zu** | **4 – stimme ich**  **eher nicht zu** | **5 – stimme ich größtenteils nicht zu** | **6 – stimme ich**  **überhaupt nicht zu** |
| Die Lernziele waren klar definiert | **☐** | **☐** | **☐** | **☐** | **☐** | **☐** |
| Die Lerninhalte wurden angemessen veranschaulicht | **☐** | **☐** | **☐** | **☐** | **☐** | **☐** |
| Der behandelte Stoff knüpfte an meinen bisherigen Wissensstand an | **☐** | **☐** | **☐** | **☐** | **☐** | **☐** |
| Der Kurs folgte einem klar erkennbaren Konzept (roter Faden) | **☐** | **☐** | **☐** | **☐** | **☐** | **☐** |
| Kommiliton:innen würde ich den Besuch dieser Veranstaltung empfehlen | **☐** | **☐** | **☐** | **☐** | **☐** | **☐** |
| *Bezüglich der Lehrpersonen:* |  |  |  |  |  |  |
| Die Dozent:innen sorgten für eine wertschätzende und anregende Lernatmosphäre | **☐** | **☐** | **☐** | **☐** | **☐** | **☐** |
| Die Dozent:innen gaben konkrete Hilfestellungen | **☐** | **☐** | **☐** | **☐** | **☐** | **☐** |
| Die Dozent:innen förderten Fragen und aktive Mitarbeit | **☐** | **☐** | **☐** | **☐** | **☐** | **☐** |
| Die Dozent:innen zeigten ausgeprägtes Interesse am Lernerfolg der Studierenden | **☐** | **☐** | **☐** | **☐** | **☐** | **☐** |
| Die Dozent:innen strahlten Begeisterung für die vertretene Fachrichtung aus | **☐** | **☐** | **☐** | **☐** | **☐** | **☐** |
| Insgesamt gesehen, bin ich mit dem Beitrag der Lehrpersonen zufrieden | **☐** | **☐** | **☐** | **☐** | **☐** | **☐** |
| *Selbstreflexion:* |  |  |  |  |  |  |
| Ich habe die Lernziele erreicht | **☐** | **☐** | **☐** | **☐** | **☐** | **☐** |
| Die Kombination mehrerer Lehrveranstaltungen (Vorbereitung, Vorlesung, echtes Tumorboard und studentisches Tumorboard) war angemessen | **☐** | **☐** | **☐** | **☐** | **☐** | **☐** |
| Ich habe mich angemessen auf die Termine vorbereitet | **☐** | **☐** | **☐** | **☐** | **☐** | **☐** |
| Ich habe mich aktiv beteiligt | **☐** | **☐** | **☐** | **☐** | **☐** | **☐** |
| Ich habe den Umgang miteinander als respektvoll empfunden | **☐** | **☐** | **☐** | **☐** | **☐** | **☐** |
| Insgesamt gesehen, bin ich mit dem Verhalten der meisten Teilnehmenden zufrieden | **☐** | **☐** | **☐** | **☐** | **☐** | **☐** |
| Mein Arbeitsaufwand war, verglichen mit anderen Lehrveranstaltungen, hoch | **☐** | **☐** | **☐** | **☐** | **☐** | **☐** |
| Die Lehrveranstaltungen förderten mein Interesse am Thema | **☐** | **☐** | **☐** | **☐** | **☐** | **☐** |
| Meinen Erkenntnisgewinn durch die Lehrveranstaltungen schätze ich hoch ein | **☐** | **☐** | **☐** | **☐** | **☐** | **☐** |
| Mein Gesamteindruck: | **☐** | **☐** | **☐** | **☐** | **☐** | **☐** |
| Wie sehr stimmst du folgenden Aussagen zu? | **1 – stimme ich vollkommen zu** | **2 – stimme ich überwiegend zu** | **3 – stimme ich**  **eher zu** | **4 – stimme ich**  **eher nicht zu** | **5 – stimme ich größtenteils nicht zu** | **6 – stimme ich**  **überhaupt nicht zu** |
| Ich halte digitales Lernen in diesem Kurs für effektiv. | **☐** | **☐** | **☐** | **☐** | **☐** | **☐** |
| Ich hätte mehr Hilfestellung gebraucht. | **☐** | **☐** | **☐** | **☐** | **☐** | **☐** |
| Die bereitgestellten Materialien waren zu viel. | **☐** | **☐** | **☐** | **☐** | **☐** | **☐** |
| Ich fühlte mich überfordert. | **☐** | **☐** | **☐** | **☐** | **☐** | **☐** |
| Ich bin zufrieden mit dem Ablauf des Kurses. | **☐** | **☐** | **☐** | **☐** | **☐** | **☐** |

Was ich sonst noch sagen wollte (Lob, Kritik, Verbesserungsvorschläge):

__________________________________________________________________________________________________________________________________________________________________________________________________________________________________________________________________________________________________________________________________________________________________________________________________________________________
