# Supplementary material for: Development and implementation of a student tumor board as a teaching format for medical students
Source: J Cancer Res Clin Oncol. 2023 Sep 12;149(17):16087–96. doi: 10.1007/s00432-023-05336-3 (PMC10620267; doi:10.1007/s00432-023-05336-3)
Supplement: Supplementary file 2 — Supplementary file2 (DOCX 32 KB) [file 432_2023_5336_MOESM2_ESM.docx]

# EVALUATION: STUDENT TUMOR BOARD

Before the course

| Gender | | | | | | ☐m ☐w ☐d | | | | | | |
| --- | --- | --- | --- | --- | --- | --- | --- | --- | --- | --- | --- | --- |
| Age | | | | | | ________years | | | | | | |
| Subject Semester | | | | | | ________Semester | | | | | | |
| I booked the course because *(multiple choice)*:  ☐ there are many C points here  ☐ the course reflects my field of interest  ☐ I learn communication strategies  ☐ it is a presence event  ☐ these skills are not promoted elsewhere  ☐ it is an interactive event  I know the lecturers. | | | | | | | | | | | | |
| What do you expect from the course?  _________________________________________________________________________  _________________________________________________________________________ | | | | | | | | | | | | |
| What would be a no-go in this course?  _________________________________________________________________________ | | | | | | | | | | | | |
| How much time do you expect to invest each week to prepare for this course? *(hours)* | | | | | | | | | | | | |
| ☐ 0 | **☐ 1** | **☐ 2** | | **☐ 3** | | | **☐ 4** | | **☐ 5** | | **☐ 6** | |
| I have attended a tumor board before | | | | | | | **☐ yes ☐ no** | | | | | |
| Please assess your prior knowledge of the subjects: | | | **1 - very good knowledge** | | **2 - good knowledge** | | | **3 - satisfactory** | **4 - sufficient** | **5 - poor** | | **6 - insufficient** |
| Neurology | | | **☐** | | **☐** | | | **☐** | **☐** | **☐** | | **☐** |
| Neurosurgery | | | **☐** | | **☐** | | | **☐** | **☐** | **☐** | | **☐** |
| Radiotherapy | | | **☐** | | **☐** | | | **☐** | **☐** | **☐** | | **☐** |
| Oncology | | | **☐** | | **☐** | | | **☐** | **☐** | **☐** | | **☐** |
| Radiology | | | **☐** | | **☐** | | | **☐** | **☐** | **☐** | | **☐** |
| Nuclear Medicine | | | **☐** | | **☐** | | | **☐** | **☐** | **☐** | | **☐** |

| Even before the course... | 1 - I completely agree | 2 - I mostly agree | 3 - I agree  rather to | 4 - I agree  rather not too | 5 - I agree for the most part  not too | 6 - I agree at all  not too |
| --- | --- | --- | --- | --- | --- | --- |
| I was able to classify the term "tumor board". | **☐** | **☐** | **☐** | **☐** | **☐** | **☐** |
| I was able to explain the goal and procedure of an interdisciplinary tumor conference. | **☐** | **☐** | **☐** | **☐** | **☐** | **☐** |
| I was able to evaluate the advantages and disadvantages of a tumor conference. | **☐** | **☐** | **☐** | **☐** | **☐** | **☐** |
| I was able to explain the basics of neuro-oncological entities. | **☐** | **☐** | **☐** | **☐** | **☐** | **☐** |
| Was I able to describe interdisciplinary therapeutic approaches to CNS neoplasms | **☐** | **☐** | **☐** | **☐** | **☐** | **☐** |
| I was able to discuss cases in an interdisciplinary way | **☐** | **☐** | **☐** | **☐** | **☐** | **☐** |
| Did I communicate effectively | **☐** | **☐** | **☐** | **☐** | **☐** | **☐** |
| Have I considered ethical aspects in medical decisions | **☐** | **☐** | **☐** | **☐** | **☐** | **☐** |
| I was able to explain a consensus decision in a professional and patient-friendly manner | **☐** | **☐** | **☐** | **☐** | **☐** | **☐** |
| Did I have interest in the participating subjects (radiotherapy, neurosurgery, oncology, pathology, radiology). | **☐** | **☐** | **☐** | **☐** | **☐** | **☐** |

# EVALUATION: STUDENT TUMOR BOARD

After the course

| Gender | | | | | | ☐ m ☐ w ☐ d | | | | | | |
| --- | --- | --- | --- | --- | --- | --- | --- | --- | --- | --- | --- | --- |
| Age | | | | | | ________years | | | | | | |
| Subject Semester | | | | | | ________Semester | | | | | | |
| What were the expectations of the course?  _________________________________________________________________________  _________________________________________________________________________ | | | | | | | | | | | | |
| What expectations have not been met?  _________________________________________________________________________ | | | | | | | | | | | | |
| What was a no-go in this course?  _________________________________________________________________________ | | | | | | | | | | | | |
| Approximately how much time did you spend each week preparing for this course? *(hours)* | | | | | | | | | | | | |
| ☐ 0 | **☐ 1** | **☐ 2** | | **☐ 3** | | | **☐ 4** | | **☐ 5** | | **☐ 6** | |
| What subject did you spend the most time on?  ___________________________________________________________________ | | | | | | | | | | | | |
| Please assess your knowledge of the subjects: | | | **1 - very good knowledge** | | **2 - good knowledge** | | | **3 - satisfactory** | **4 - sufficient** | **5 - poor** | | **6 - insufficient** |
| Neurology | | | **☐** | | **☐** | | | **☐** | **☐** | **☐** | | **☐** |
| Neurosurgery | | | **☐** | | **☐** | | | **☐** | **☐** | **☐** | | **☐** |
| Radiotherapy | | | **☐** | | **☐** | | | **☐** | **☐** | **☐** | | **☐** |
| Oncology | | | **☐** | | **☐** | | | **☐** | **☐** | **☐** | | **☐** |
| Radiology | | | **☐** | | **☐** | | | **☐** | **☐** | **☐** | | **☐** |
| Nuclear Medicine | | | **☐** | | **☐** | | | **☐** | **☐** | **☐** | | **☐** |
|  | | |  | |  | | |  |  |  | |  |

| After the course... | 1 - I completely agree | 2 - I mostly agree | 3 - I agree  rather to | 4 - I agree  rather not too | 5 - I agree for the most part  not too | 6 - I agree at all  not too |
| --- | --- | --- | --- | --- | --- | --- |
| I can classify the term "tumor board". | **☐** | **☐** | **☐** | **☐** | **☐** | **☐** |
| I can explain the aim and procedure of an interdisciplinary tumor conference. | **☐** | **☐** | **☐** | **☐** | **☐** | **☐** |
| I can evaluate the advantages and disadvantages of a tumor conference. | **☐** | **☐** | **☐** | **☐** | **☐** | **☐** |
| I can explain the basics of neuro-oncological entities. | **☐** | **☐** | **☐** | **☐** | **☐** | **☐** |
| Can I describe interdisciplinary therapeutic approaches to CNS neoplasms. | **☐** | **☐** | **☐** | **☐** | **☐** | **☐** |
| Can I discuss cases interdisciplinary | **☐** | **☐** | **☐** | **☐** | **☐** | **☐** |
| I can communicate more effectively | **☐** | **☐** | **☐** | **☐** | **☐** | **☐** |
| I tend to take ethical aspects into account in medical decisions. | **☐** | **☐** | **☐** | **☐** | **☐** | **☐** |
| I can explain a consensus decision in a professional and patient-friendly manner | **☐** | **☐** | **☐** | **☐** | **☐** | **☐** |
| Do I have interest in the participating subjects (radiotherapy, neurosurgery, oncology, pathology, radiology). | **☐** | **☐** | **☐** | **☐** | **☐** | **☐** |
| How much do you agree with the following statements? | **1 - I completely agree** | **2 - I mostly agree** | **3 - I agree**  **rather to** | **4 - I agree**  **rather not too** | **5 - I disagree for the most part** | **6 - I agree**  **not at all** |
| The learning objectives were clearly defined | **☐** | **☐** | **☐** | **☐** | **☐** | **☐** |
| The learning content was appropriately illustrated | **☐** | **☐** | **☐** | **☐** | **☐** | **☐** |
| The material covered tied in with my previous level of knowledge | **☐** | **☐** | **☐** | **☐** | **☐** | **☐** |
| The course followed a clearly recognizable concept (red thread) | **☐** | **☐** | **☐** | **☐** | **☐** | **☐** |
| I would recommend the attendance of this event to fellow students | **☐** | **☐** | **☐** | **☐** | **☐** | **☐** |
| *Regarding teachers:* |  |  |  |  |  |  |
| The instructors provided an appreciative and stimulating learning atmosphere. | **☐** | **☐** | **☐** | **☐** | **☐** | **☐** |
| The lecturers provided concrete assistance | **☐** | **☐** | **☐** | **☐** | **☐** | **☐** |
| The instructors encouraged questions and active participation. | **☐** | **☐** | **☐** | **☐** | **☐** | **☐** |
| The lecturers showed a strong interest in the learning success of the students. | **☐** | **☐** | **☐** | **☐** | **☐** | **☐** |
| The lecturers radiated enthusiasm for the represented discipline | **☐** | **☐** | **☐** | **☐** | **☐** | **☐** |
| Overall, I am satisfied with the contribution of the teachers | **☐** | **☐** | **☐** | **☐** | **☐** | **☐** |
| *Self-reflection:* |  |  |  |  |  |  |
| I have achieved the learning objectives | **☐** | **☐** | **☐** | **☐** | **☐** | **☐** |
| The combination of multiple courses (preparation, lecture, real tumor board, and student tumor board) was appropriate | **☐** | **☐** | **☐** | **☐** | **☐** | **☐** |
| I have prepared adequately for the appointments | **☐** | **☐** | **☐** | **☐** | **☐** | **☐** |
| I have actively participated | **☐** | **☐** | **☐** | **☐** | **☐** | **☐** |
| I have found the interaction with each other to be respectful | **☐** | **☐** | **☐** | **☐** | **☐** | **☐** |
| Overall, I am satisfied with the behavior of most participants | **☐** | **☐** | **☐** | **☐** | **☐** | **☐** |
| My workload, compared to other courses, was high | **☐** | **☐** | **☐** | **☐** | **☐** | **☐** |
| The courses furthered my interest in the subject of | **☐** | **☐** | **☐** | **☐** | **☐** | **☐** |
| I rate my knowledge gain from the courses highly | **☐** | **☐** | **☐** | **☐** | **☐** | **☐** |
| My overall impression: | **☐** | **☐** | **☐** | **☐** | **☐** | **☐** |
| How much do you agree with the following statements? | **1 - I completely agree** | **2 - I mostly agree** | **3 - I agree**  **rather to** | **4 - I agree**  **rather not too** | **5 - I disagree for the most part** | **6 - I agree**  **not at all** |
| I think digital learning is effective in this course. | **☐** | **☐** | **☐** | **☐** | **☐** | **☐** |
| I could have used more assistance. | **☐** | **☐** | **☐** | **☐** | **☐** | **☐** |
| The materials provided were too much. | **☐** | **☐** | **☐** | **☐** | **☐** | **☐** |
| I felt overwhelmed. | **☐** | **☐** | **☐** | **☐** | **☐** | **☐** |
| I am satisfied with the way the course went. | **☐** | **☐** | **☐** | **☐** | **☐** | **☐** |

What else I wanted to say (praise, criticism, suggestions for improvement):

__________________________________________________________________________________________________________________________________________________________________________________________________________________________________________________________________________________________________________________________________________________________________________________________________________________________
